# Breastfeeding, breast milk and viruses

**DOI:** 10.1186/1472-6874-7-17

**Published:** 2007-10-08

**Authors:** James S Lawson, Joy Heads, Wendy K Glenn, Noel J Whitaker

**Affiliations:** 1 School of Public Health and Community Medicine, University of New South Wales, Sydney, Australia; 2 Royal Hospital for Women, Sydney, Australia; 3 School of Biotechnology and Biomolecular Science, University of New South Wales, Sydney, Australia

## Abstract

**Background:**

There is seemingly consistent and compelling evidence that there is no association between breastfeeding and breast cancer. An assumption follows that milk borne viruses cannot be associated with human breast cancer.

We challenge this evidence because past breastfeeding studies did not determine "exposure" of newborn infants to colostrum and breast milk.

**Methods:**

We conducted a prospective review of 100 consecutive births of infants in the same centre to determine the proportion of newborn infants who were "exposed" to colostrum or breast milk, as distinct from being fully breast fed. We also report a review of the breastfeeding practices of mothers of over 87,000 newborn infants in the Australian State of New South Wales.

This study was approved by the Human Research Ethics Committee of the University of New South Wales (Sydney, Australia). Approval 05063, 29 September 2005.

**Results:**

Virtually all (97 of 100) newborn infants in this centre were "exposed" to colostrum or breast milk whether or not they were fully breast fed. Between 82.2% to 98.7% of 87,000 newborn infants were "exposed" to colostrum or breast milk.

**Conclusion:**

In some Western communities there is near universal exposure of new born infants to colostrum and breast milk. Accordingly it is possible for the transmission of human milk borne viruses. This is contrary to the widespread assumption that human milk borne viruses cannot be associated with breast cancer.

## Background

Human immunodeficiency virus (HIV), cytomegalovirus (CMV), human T cell leukaemia virus (HTLV) and other viruses may be transmitted by human milk and cause infections and disease [1]. In animals, viruses, such as the mouse mammary tumor virus (MMTV), are transmitted by mouse milk to new born mouse pups and are a proven cause of mouse mammary tumours [2].

Accordingly, the transmission of milk born viruses which may have a role in human breast cancer has long been postulated. However, there is extensive and consistent epidemiological evidence, which indicates that breastfeeding – and hence the opportunity to transfer viruses via human mothers milk to infants, is not related to breast cancer [3,4]. There also appears to be no association between risk of breast cancer and breastfeeding by daughters whose mothers had breast cancer [5].

There is substantial, but not conclusive, evidence which suggests that a virus, virtually identical with mouse mammary tumor virus and additional viruses including high risk human papilloma viruses (HPVs) and Epstein-Barr virus (EBV) may have roles in the etiology of breast cancer [2]. However, with respect to breast cancer etiology, the transmission routes for these viruses is not known. For this reason, we re-examined the evidence relating to breastfeeding and breast cancer. We discovered there is a problem with the epidemiological studies (see meta analyses by Beral et al and Martin et al relating to breastfeeding [3,4]). There has been almost universal use of the seemingly simple question "did you breast feed your baby and if so, for how long?" This question does not necessarily determine if a newborn infant was "exposed" to colostrum or breast milk, whether or not the mother subsequently established breast feeding. In addition, surveys in which this question was asked were often conducted many years after the event [4]. Labbock et al realised that lack of precision and consistency in the definition of breastfeeding had led to misinterpretation of data and problems with comparability between studies [6]. In neither of the recent meta-analyses was this crucial definitional issue considered in detail [3,4].

Therefore we hypothesised (i) that a high proportion of newborn infants may be "exposed" to colostrum or breast milk whether or not they were breast fed, (ii) if this hypothesis was shown to be true, that viruses could be transmitted to new born human infants. We tested the first hypothesis in a prospective study and reviewed existing data concerning exposure of newborns to colostrum and breast milk in Australia.

## Methods

We conducted a prospective review of 100 consecutive births of infants, which had all been at the same location during 2006 (Royal Hospital for Women, Sydney, Australia). We used a standard definition of "ever breastfed" as "*those infants who have been put to the breast, if only once, and includes infants who have received expressed breast milk but have never been put to the breast"*. The proportion of "ever breastfed infants" who were "exposed" to colostrum or breast milk was determined.

We reviewed the published data on breastfeeding practices in the Australian State of New South Wales (NSW) [7]. Each year there are approx. 87,000 births in NSW. A record is kept by midwives of the breastfeeding practices of mothers of each new born infant (there is a near 100% response rate). The standard definition of "ever breastfed" as defined above, was used for the collection of this data [7].

### Statistics

One sample Chi square was used to measure the proportion of newborn infants that were exposed to colostrum or breast milk.

## Results

Ninety seven (97%) of the 100 infants consecutively born at the Royal Hospital for Women during April and May of 2006, were "ever breastfed" (Table 1). Each of the mothers of the 3 infants who were not "ever breast fed" had indicated they did not wish to breast feed (Table 1). All 100 newborn infants "nuzzled" their mother's nipple and breast, including the 3 who were not exposed to colostrum or breast milk.

**Table 1:** Proportion of newborn infants exposed to colostrum or breast milk

Number of mothers	Number of infants exposed	Number of infants not exposed	Significance at 95% level
100	97	3	p = 0.000

The percentage of the approx 87,000 infants born during 2001 in NSW who were "ever breastfed" averaged 90.2%. This percentage varied from a high of 98.7% to a low of 82.2% for infants born of mothers from communities of high compared to lower socio-economic status respectively. "Ever breastfed" rates also varied according to the age and education of the mother. 84.5% of mothers under the age of 25 years and 90.4% of mothers aged 25 years or over "ever breast fed". 86.8% of mothers with primary and secondary school education and 96.1% of mothers with tertiary education (University and college levels) "ever breast fed". These percentages fell dramatically during the first year of life of these infants, 54.2% were receiving "any breast milk" at 4 months after birth, 42.5% at 6 months and 18.1% at 12 months.

## Discussion

The proportion of newborn infants "exposed" to colostrum or breast milk during recent years in NSW, Australia is far higher than that reported in any of the studies of breastfeeding practices in other Western countries. This observation suggests that there is a possibility of colostrum or milk borne transmission of viruses to virtually all infants in these Australian communities. While these findings cannot be generalised to other countries and communities, the accepted opinion that there is no association between breastfeeding and breast cancer may not be true.

With respect to studies which have sought to determine whether or not the *duration *of breastfeeding is associated with risk of breast cancer, it is likely that the data is sufficiently reliable to allow conclusions to be drawn [3,4]. This is because the data required is much less specific than that required to determine any early life exposure to breast colostrum or breast milk. These many studies have shown there is no increased risk of breast cancer associated with prolonged breastfeeding [3,4]. It follows that there is unlikely to be a "dose response" based on breastfeeding for months, associated with any milk transmitted virus or other agent that may be associated with breast cancer.

With the important exception of HIV, data concerning human milk borne viruses is very limited. HIV is the same family of retroviruses as MMTV and is therefore relevant. The viral load of HIV in human milk and colostrum is highest in the newborn period [8]. However human milk transmission of HIV is a complex phenomenon [9].

There is sparse evidence which suggests MMTV-like genetic material may be present in human milk. Moore et al have observed by electron microscopy MMTV like images in human milk [10]. Ford has made preliminary identification of MMTV-like DNA sequences in human milk [11]. As HIVs are known to be transmitted in high concentrations by human milk to newborn human infants and to a lesser extent in older infants, it is theoretically possible for MMTV to be also transmitted by only a brief exposure to colostrum and breast milk. There are no published studies of human milk transmission of MMTV.

## Conclusion

In conclusion, we have shown that the first hypothesis, that a high proportion of newborn infants may be "exposed" to colostrum or breast milk whether or not they were breast fed, is likely to be correct. The second hypothesis that viruses such as MMTV could be transmitted to newborn human infants, remains to be tested. In our view, this second hypothesis is biologically feasible.

## Competing interests

The author(s) declare that they have no competing interests.

## Authors' contributions

JL conceptualised and organised the investigation, analysed the data and drafted the manuscript.

JH organised the investigation and drafted the manuscript. WG conceptualised and organised the investigation. NW analysed the data and drafted the manuscript.

All authors read and approved the final manuscript.

## Pre-publication history

The pre-publication history for this paper can be accessed here:


